# Effectiveness of Faith-Based Interventions on the Rate of Discharged Against Medical Advice in Tertiary Newborn Units in Nigeria: A Protocol for an Open Label Randomized Control Trial

**DOI:** 10.3389/fpubh.2021.788383

**Published:** 2022-02-01

**Authors:** Michael Abel Alao, Olayinka Rasheed Ibrahim, Babatunde Oluwatosin Ogunbosi, Emmanuel Okechukwu Nna, Peter Olamakinde Olapegba

**Affiliations:** ^1^Department of Paediatrics, University College Hospital Ibadan, Ibadan, Nigeria; ^2^Department of Paediatrics, Federal Medical Centre, Katsina, Nigeria; ^3^The Molecular Pathology Institute, Enugu, Nigeria; ^4^Department of Psychology, University of Ibadan, Ibadan, Nigeria

**Keywords:** discharged against medical advice, religion, faith-based interventions, Nigeria, neonates, newborn, randomized control trial

## Abstract

**Background:**

Discharged against medical advice (DAMA) is a risk factor that often leads to adverse outcomes and hospital readmissions in neonatal units. A few studies have shown that spiritual/faith-based interventions (FBIs) tend to have a lower incidence of DAMA compared with public hospitals. Perhaps, a holistic approach to patient care that addresses the spiritual needs, the soul and the body component of a being in this setting may account for the observed lower incidence of DAMA. Limited randomized control trials (RCTs) exist on FBIs with regard to DAMA in the published literature. This study seeks to compare the effectiveness of FBI, social support, religiosity, and types of FBI on neonatal DAMA against standard of care in tertiary hospitals in Nigeria.

**Methods:**

This RCT will be conducted in two public tertiary teaching hospitals in two of the six geopolitical zones in Nigeria. The sociodemographic and clinical details of all patients admitted to the neonatal wards during the study period will be documented. Study participants will be selected through a multistage sampling technique. Subjects will be randomized and allocated to treatment and control arms having the established baseline measure of social support and religiosity. Ethical approval was obtained from the State Research Ethics Review Committee. A written informed consent will be obtained from the parents/caregivers prior to patient enrolment. The study will be conducted in line with the Declaration of Hesinki 2000. Appropriate statistical tools will be used for data collection and analysis.

**Discussion:**

The outcome of this analysis will give insights into the effectiveness of FBI on DAMA. It will also predict the effect of the mediators of parents/caregivers' religiosity, spirituality, forms of FBI, the religious sect of parents/caregivers, and social support on the rate of DAMA on neonatal admission in tertiary hospitals in Nigeria. This could help Public Health Institutions and Governments make decisions about the determinants of neonatal DAMA and how to mitigate such outcomes. It is hoped that the evidence from this study may guide policy formulation and guidelines on enhancing hospital retention of sick neonates until they are fit for discharge.

**Trial Registration:**

This study was registered at the Pan Africa Clinical Trial Registry (PACTR202102670906630).

## Background and Rationale

Approximately 130 million babies are born annually, about 4 million of these newborns in low- and middle-income countries (LMICs) do not survive beyond the neonatal period (1). More worrisome is the increasing contribution of childhood mortality from the neonatal death. This narrative is not different in Nigeria where neonatal mortality represents one of the highest in the world, only behind India (2).

The reasons for this trend are multifactorial. They vary from ignorance, harsh healthcare workers altitude to caregivers/parents, poverty, the lack of access to good healthcare, the need for spiritual support at a very trying time, and discharged against medical advice (DAMA) (3–7). It is, therefore, important that those who get to a health service be treated holistically, providing physical, psychological, and spiritual support to enable them complete treatment until when discharge is recommended by the treating clinicians (3–5).

Discharged against medical advice occurs when an in-patient decides to leave the hospital before discharge is recommended by the treating clinicians or physicians. DAMA poses serious clinical, ethical, and legal challenges to the individual physician as well as to the hospital (1, 6–9). The DAMA prevalence has been shown to vary depending on geographical areas and the study population. The rate of DAMA compares inversely with the socioeconomic status: a relatively lower rate was observed at a hospital serving primarily middle- and upper-class populations, whereas a higher rate was observed at a hospital serving disadvantaged urban areas (9–13). The pediatric age group, and especially the newborns are at a greatest risk for DAMA in Nigeria from the published literature (9–11).

Discharged against medical advice has been shown to increase the risk of adverse outcomes ranging from medical complications requiring readmission to death (6, 8). Associated outcomes also include higher morbidity, increased mortality, longer hospital stays, and higher costs of treatment when readmitted (10, 11).

More challenging with DAMA is the ethical issues in neonates as they have the least autonomy to participate in their health decision. The parents/caregivers entrusted with the right of decision-making often fail this vulnerable population. The very sick babies with a risk for residual long-term outcomes, higher risk for mortality, and foreseeable future of being a burden are often thought of dispensing with by the parents/caregivers, and hence there is a request for DAMA. This poses a great challenge to the managing physician on maintaining a balance between the parents/caregivers autonomy against the fiduciary role of the physician (4, 6, 8).

A few observational studies have shown lower incidences of DAMA in faith-based hospitals where spiritual leaders actively participate in patient care (14–20). A holistic approach to patients' care that addresses the spiritual needs, the soul, and the body component of a being in this setting may account for the observed lower incidence of DAMA. However, it may not be sufficient to explain the lower incidence of DAMA with just the involvement of the spiritual leader in care, the literature has suggested that a number of variables can interact to influence decision-making. In this instance, perceived social support of parents/caregivers of newborns as well as their levels of religiosity may significantly influence the decision to engage in DAMA (20–22). Limited randomized control trial (RCT) studies on the effectiveness of faith-based intervention (FBI) are available in the published literature.

This protocol provides a workflow for an open label randomized clinical trial to evaluate the effectiveness of spiritual/FBI intervention on hospital retention of neonates compared with standard of care in tertiary hospitals. It also includes secondary outcomes such as patients' clinical outcome, parents/caregivers' satisfaction with intervention, and their desire to see the intervention established as routine care for newborns in a public tertiary hospital.

## Objectives

### General Objective

The general objective is to determine the effectiveness of religion intervention rates of DAMA in neonates.

### Specific Objectives

To determine:

1. a comparison of the effectiveness of religion intervention with standard of care on newborn rates of DAMA.
2. the association in case of between clinical and sociodemographic characteristic of patients on the rates of DAMA.
3. the delineation of the reasons for DAMA among neonates in Nigeria.
4. a model for a prediction of the rates of DAMA in neonates using explanatory variables (reasons) for DAMA.
5. the effect of parents/caregivers religiosity, spirituality, types of FBI, the religious sect of parents/caregivers, and social support on the outcomes of DAMA or hospital retention till discharge.

### Trial Design

#### Intervention Assignment

This will be an open label, parallel RCT.

Simple randomization using a randomization table created by a computer software program would be used for randomization.

Allocation sequence would be concealed in sealed opaque envelopes.

## Methods: Participants, Interventions, and Outcomes

### Study Setting

This study is a multicenter study involving two public tertiary hospitals located in two of the six geopolitical zones/regions in Nigeria (23, 24). The selected tertiary hospital includes University College Hospital Ibadan and The Federal Medical Center Katsina, Katsina state as shown in Figure 2.

### Eligibility Criteria

#### Inclusion Criteria

1. All newborns admitted into selected public hospitals whose parents/caregivers gave their consent to participate in this study.

#### Exclusion Criteria

1. Babies whose parents/caregivers fail to give consent for this study.
2. Babies were taken into custody by institutions such as motherless home or by government agencies for legal reasons.

### Who Will Take Informed Consent?

Prior to recruitment, the research would be explained to parents/caregivers by the investigator who will obtain a written informed consent. Parents/caregivers will be informed of their freedom to refuse to take part in this study without any negative consequences to them or their wards in the course of treatment.

### Additional Consent Provisions for the Collection and Use of Participant Data and Biological Specimens

Additional consent would be obtained for data availability for secondary analysis and for ancillary studies in the future.

### Interventions

#### Explanation for the Choice of Comparators

The comparator will provide standard of care for all babies admitted to a newborn unit. The active treatment of control arm is in line with basic principles of medical ethics for RCT.

#### Intervention Description

There will be two arms of this study; one arm (the experimental arm) will receive a FBI. The FBI will involve religious counseling encouraging the caregivers/parents to stay in the hospital until their baby is medically discharged. This will also involve offering prayers and reading of holy books for the babies recovery. Each participants will have two to three sessions of the FBI with each session lasting 20–30 min.

The control arm will be exposed to standard of care observed for all babies admitted to a newborn unit.

### Criteria for Discontinuing or Modifying Allocated Interventions

Participant who also wishes to exit study after due counseling.

### Strategies to Improve Adherence to Interventions

Prior to recruitment, parents/caregivers of study participants will be educated on the study in order to gain their cooperation.

### Relevant Concomitant Care Permitted or Prohibited During the Trial

Both the experimental and control group will have access to the same treatment except for the intervention in the experimental arm.

### Provisions for Post-trial Care

The outcome of measure is a one-time off measure and would not require a follow-up.

### Outcomes

#### The Primary Outcome

1. The primary outcome of this study is the retention rate of sick newborns with the FBI in public tertiary hospitals in Nigeria compared with the standard of care.

#### Secondary Outcomes

1. The secondary outcomes are the reasons and determinants of DAMA among neonates in tertiary hospitals in Nigeria.

The effect of parents/caregivers religiosity, spirituality, types of FBI, the sect of parents/caregivers, and social support on the outcomes of DAMA or hospital retention in neonatal admission.

1. Patients' clinical outcome.
2. Parents/caregivers' satisfaction with intervention and their desire to see the intervention established as routine care for newborns in a public tertiary hospital.

### Participant Timeline

#### Sample Size

The details of participant timeline is shown in Table 1. The sample size required for this study was determined using the Raosoft sample size calculator (http://www.raosoft.com/samplesize.html) for single proportion with estimated 50% prevalence of DAMA. A sample size of 359 has 80% power to detect the prevalence of cerebral malaria at an alpha level of probability 0.05.

**Table 1:** Participant timeline (25).

	**STUDY PERIOD**							
	**Enrolment**	**Allocation**	**Post-allocation**					**Close-out**
**Timepoint**	***-t_1_***	**0**	***12hs***			***72hrs***		***t_x_***
**Enrolment:**								
Eligibility screen	X							
Informed consent	X							
*Administration of research screening tools*	X							
*Clinical history and examination*	X					X		
Allocation		X						
**Interventions:**								
*[Intervention A]*			X 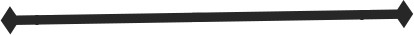 X				
*[Intervention B]*			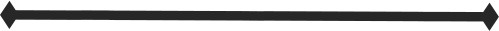				
**Assessments:**								
*[List outcome variables]*								X

#### Recruitment

**Figure 1:**
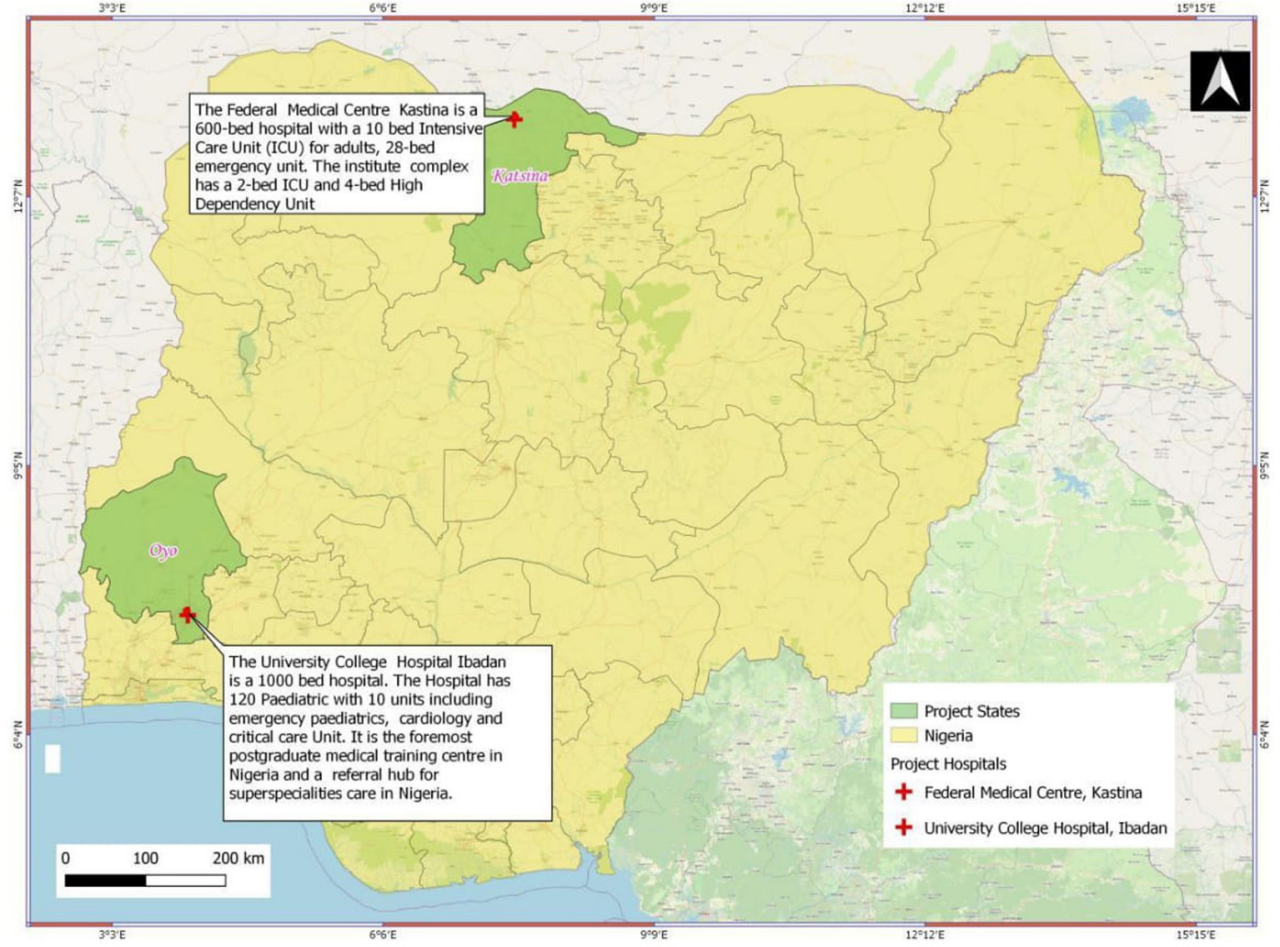
Study participant flow chart.

The details of patients' recruitment are shown in the study participant flow chart in Figure 1.

**Figure 2:**
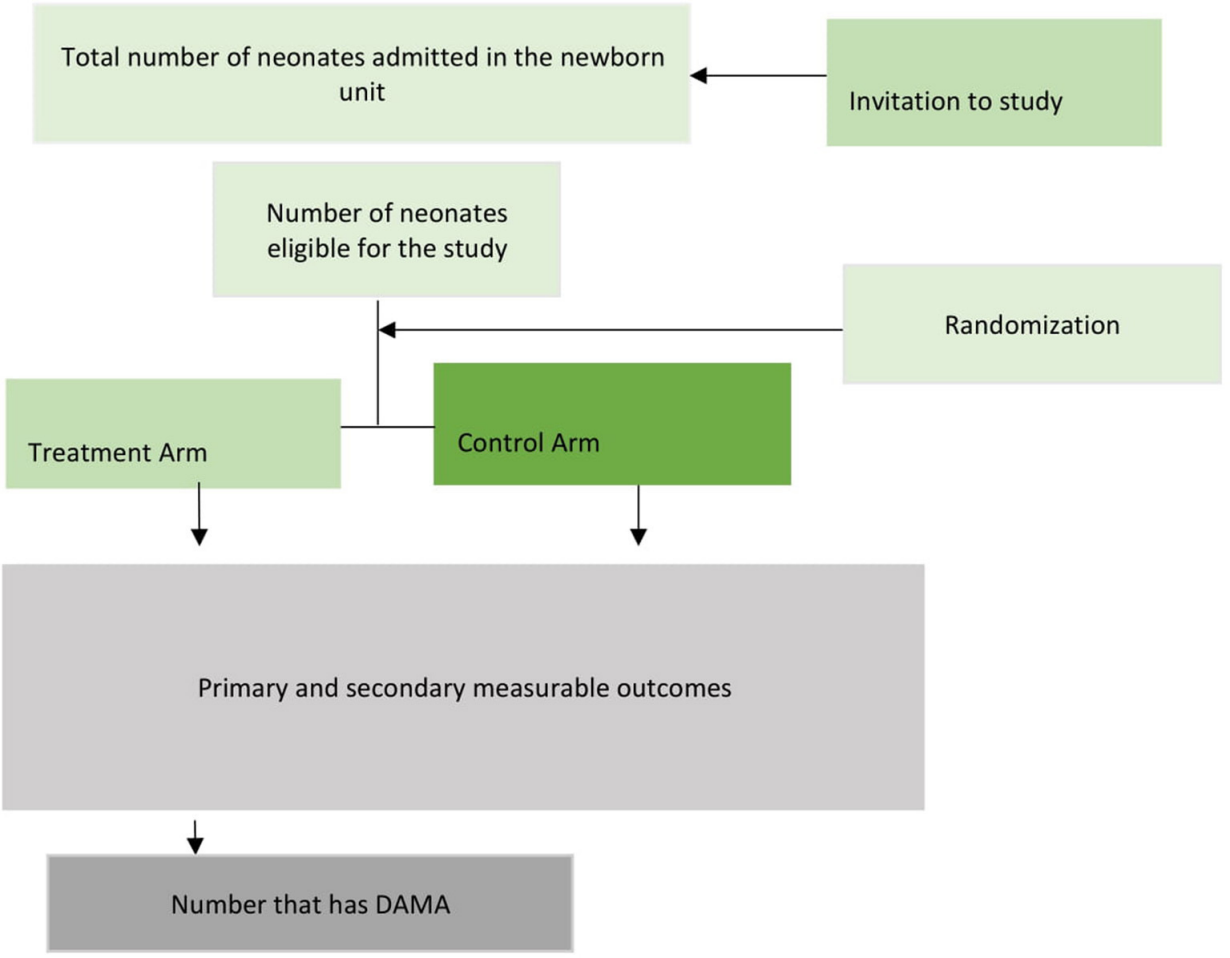
Map of the study centers in Nigeria.

*Invitation*: the caregivers/parents of eligible patients will be verbally invited during their hospital admission.

*Eligibility*: subjects will be assessed based on the eligibility criteria enumerated earlier.

*Enrolment*: eligible subjects will be enrolled into this study after giving a written informed consent.

*Informed consent*: parents and caregivers will give a written informed consent during enrolment. This will be signed by Principal Investigator, the parent/caregiver, and a witness. A copy of informed consent will be retained by the parent, while a copy will be kept in the patient's file.

#### Sampling Technique

*In stage I*, the list of tertiary hospitals in Nigeria will form the sample frame for this study. A random number will be allocated to each center. Two institutions will be randomly selected from this list.

*In Stage II*, an allocation sequence for the two arms will be generated using simple randomization from GraphPad Prism version 9. In the selected hospitals, consecutive neonates whose parents/caregivers give an informed consent would be allocated to one of the two arms based on the allocation sequence.

### Allocation

Allocation will be done by simple randomization using the random numbers generated from GraphPad Prism (version 9) for this study.

1. Arm A: The intervention for FBI.
2. Arm B: The standard of care for neonates admitted into the unit born units of selected hospital.

Subjects will be allocated to two arms of this study in parallel (concurrent): FBI (Arm A) and standard of care (Arm B) based on the randomization process.

### Assignment of Interventions: Allocation

#### Sequence Generation

A random number will be generated using simple randomization from GraphPad Prism version 9 for consecutive patients being enrolled for this study.

#### Concealment Mechanism

This study will be an open label (masking not used).

#### Implementation

A record officer will be responsible for generating a random number and its allocation using GraphPad Prism version 9.

### Assignment of Interventions: Blinding

#### Who Will Be Blinded

This is an open label trial. The intervention and comparator will not be concealed. Both the investigator and the subject will be aware of what intervention they would receive.

#### Procedure for Unblinding if Needed

This study will be an open label (masking not used).

### Data Collection and Management

#### Plans for Assessment and Collection of Outcomes

Training will be held for the researcher prior to the commencement of the trial *via* online zoom meeting. The validated questionnaire, the trial protocol, religiosity, spirituality, and social scales would be tested. Experience on understanding the tools and the ease of administration of the tools would be assessed. The observation from the training will be incorporated into the study instruments to improve the data entry and address other observed limitations.

#### Plans to Promote Participant Retention and Complete Follow-Up

This study's outcome would be measured at a single time point, with no follow up.

### Data Management

The data obtained from this study will be entered into a password and encrypted institutional Red Cap database. Only specific individuals from the collaborating centers will be given access to the database. The data from all the centers will be de-identified and managed through a secured code.

### Confidentiality

All information collected in this study will be given code numbers, and no name will be recorded. This cannot be linked to the patients, parents, or a care provider in any way. Identifier will not be used in any publication or reports from this study.

#### Plans for Collection, Laboratory Evaluation, and Storage of Biological Specimens for Genetic or Molecular Analysis in This Trial/Future Use

This trial has no intention to collect the biological samples for a genetic study.

### Statistical Methods for Primary and Secondary Outcomes

Data from this study will be analyzed in GraphPad Prism 9 (GraphPad Software, San Diego, CA, USA). The appropriate descriptive statistics will be used to present the sociodemographic characteristics of study participants. The comparison of categorical outcomes between the arms will be analyzed using the Chi-squared or Fisher's exact tests, as appropriate, and presented as risk differences, risk ratios, or odds ratios and 95% CIs. The values of *p* < 0.05 will be considered statistically significant for all analyses.

### Interim Analyses

Data will be analyzed at the end of this study.

#### Methods for Additional Analyses (e.g., Subgroup Analyses)

Subgroup analysis will be performed using variables such as geopolitical region, gender, socioeconomic status of parents and care providers, and the levels of education and occupation.

#### Methods in Analysis to Handle Protocol Nonadherence and Any Statistical Methods to Handle Missing Data

The result of this study will be analyzed per protocol. Missing data will be accounted and the proportion with a desirable outcome will be presented.

#### Plans to Give Access to the Full Protocol, Participant Level-Data, and Statistical Code

The protocol shall be published in a peered review journal and made publicly accessible to interested individuals or body.

Individual patient data will be de-identified and stored to be encrypted in a passworded computer. De-identified data will also be stored in highly secured cloud computing. The participant level-data set and statistical code shall be made available after following due process adhering to good ethical standard.

### Sharing Time Frame

The de-identified data will be publicly available for 2 years on the trial website.

#### Key Access Criteria

Open access to de-identified data set, which can be used for any analysis related to DAMA.

### Oversight and Monitoring

#### Composition of the Coordinating Center and Trial Steering Committee

A trial steering committee consists of the Principal Investigator, two scientific enquirers, the public enquirer, and a biostatistician. They will meet frequently to provide an oversight function for the trial conduct over the two centers in the country.

Each center will have a hospital trial group headed by a consultant pediatrician, who will be saddled with running daily events in the hospital, providing organizational support, and reporting on a weekly basis to the steering committee.

#### Composition of the Data Monitoring Committee and Its Role and Reporting Structure

The data monitoring committee consists of the Head of the Information Technology at the University College Hospital Ibadan. He will centrally manage the database. He will be supported by two assistants if he is unable to perform his duties. They will be responsible for entering the data from the UCH center to the database. The head will give access to focal persons (information technologist) at the collaborating centers in Nigeria. These individuals will be responsible for entering the data into the central database in UCH. Regular zoom meeting will be held among the group members to address pressing issue. The data monitoring committee shall be independent of the core trial committee.

### Adverse Event Reporting and Harms

The trial is asocial intervention. If any instance of abuse is reported by the participant, they will be handled on case by case bases by the steering committee.

#### Frequency and Plans for Auditing Trial Conduct

The local ethics board will monitor the progress of this trial, and all medications and update will be relayed to the body as events unfold.

#### Plans for Communicating Important Protocol Amendments to Relevant Parties (e.g., Trial Participants and Ethical Committees)

Any modification to the protocol or trial update will be communicated to the Ethical Approval bodies, Trial Registry, and any other relevant parties.

### Dissemination Plans

The outcome of this study will be communicated to participants, ethics board, and healthcare professionals. It will be published in peer-reviewed scientific journals for public access. Data will be made available to the public maintaining ethic guidance.

## Discussion

The outcome of this analysis will give insights into the effectiveness of FBI on DAMA. It will also predict the effect of the mediators of parents/caregivers religiosity, spirituality, forms of FBI, the religious sect of parents/caregivers, and social support on the rate of DAMA on neonatal admission in tertiary hospitals in Nigeria. This could help Public Health Institutions and Governments make decisions about the determinants of neonatal DAMA and how to mitigate such outcomes. It is hoped that the evidence from this study may guide policy formulation and guidelines on enhancing hospital retention of sick neonates until they are fit for discharge.

## Ethics Statement

The studies involving human participants were reviewed and approved by ethical approval was obtained from the State Research Ethics Review Committee (Ref:AD13/479/3047A) and the trial was also registered (PACTR202102670906630). Written informed consent to participate in this study was provided by the participants' legal guardian/next of kin.

## Author Contributions

MA conceived the study, led the proposal and protocol development, critically revised the protocol for important intellectual content, gave final approval, and agreed to be accountable for all aspects of the work in ensuring that questions related to the accuracy or integrity of any part of the work are appropriately investigated and resolved. OI, BO, EN, and PO contributed to the design and development of the proposal and protocol, critically revised protocol, gave final approval, and agreed to be accountable for all aspects of work ensuring integrity and accuracy. All authors read and approved the final manuscript.

## Funding

This study was currently funded by MA and the coauthors.

## Conflict of Interest

The authors declare that the research was conducted in the absence of any commercial or financial relationships that could be construed as a potential conflict of interest.

## Publisher's Note

All claims expressed in this article are solely those of the authors and do not necessarily represent those of their affiliated organizations, or those of the publisher, the editors and the reviewers. Any product that may be evaluated in this article, or claim that may be made by its manufacturer, is not guaranteed or endorsed by the publisher.

## Abbreviations

RCT: Randomized clinical trial
PACTR: Pan Africa Clinical Trial Registry
FBI: Faith-based intervention
LMIC: Low- and middle-income countries.
